# Simplified Beam Hardening Correction for Ultrafast X-ray CT Imaging of Binary Granular Mixtures

**DOI:** 10.3390/s24102964

**Published:** 2024-05-07

**Authors:** Martina Bieberle, Theodoros Nestor Papapetrou, Gregory Lecrivain, Dominic Windisch, André Bieberle, Michael Wagner, Uwe Hampel

**Affiliations:** 1 Helmholtz-Zentrum Dresden-Rossendorf, Institute of Fluid Dynamics, Bautzner Landstr. 400, 01328 Dresden, Germany; 2 Institute of Power Engineering, TUD Dresden University of Technology, Nöthnitzer Str. 69, 01062 Dresden, Germany

**Keywords:** beam hardening, computed tomography, image reconstruction, ultrafast measurement, granular media, particle mixing, rotating drum

## Abstract

Ultrafast X-ray computed tomography is an advanced imaging technique for multiphase flows. It has been used with great success for studying gas–liquid as well as gas–solid flows. Here, we apply this technique to analyze density-driven particle segregation in a rotating drum as an exemplary use case for analyzing industrial particle mixing systems. As glass particles are used as the denser of two granular species to be mixed, beam hardening artefacts occur and hamper the data analysis. In the general case of a distribution of arbitrary materials, the inverse problem of image reconstruction with energy-dependent attenuation is often ill-posed. Consequently, commonly known beam hardening correction algorithms are often quite complex. In our case, however, the number of materials is limited. We therefore propose a correction algorithm simplified by taking advantage of the known material properties, and demonstrate its ability to improve image quality and subsequent analyses significantly.

## 1. Introduction

X-ray computed tomography is well known as a medical diagnostic tool [1,2,3], but is also widely used in industrial applications and other fields for nondestructive testing [4,5,6,7]. An uncommon representative of this technique is the ultrafast X-ray computed tomography (UFXCT) [8], which is purpose-built for the investigation of transient multiphase flow phenomena, especially for those happening in opaque vessels [9,10]. As the latter are not accessible by optical measurement techniques, tomographic imaging techniques are the only non-invasive alternative. The distinctive feature of UFXCT is its high frame rate of up to 8000 cross-sectional images per second, which enables even rapidly moving or changing flow structures like particles [8], gas bubbles [9] or liquid wisps [10] to be captured. Besides this, structure and particle velocities are accessible in an optional dual plane mode [8]. However, the price to be paid for the high frame rate is a lower signal-to-noise ratio compared to common CT systems, which in turn reduces the spatial and contrast resolution of the images. Therefore, other fault effects that may reduce the image quality should be kept as low as possible. Depending on the respective object of interest, i.e., the spatial and temporal distribution of certain materials with their respective physical properties, different effects might become relevant. Here, we focus on beam hardening caused by the high fraction of glass within the measurement plane.

The scientific objective forming the background of this paper is to study binary granular mixing in a rotating drum [11], which is widely used as a reference scenario for different kinds of industrial applications of granular mixing [12], such as in the pharmaceutical, food or cement industries. Precisely, we focus on mixtures of spherical particles of the same size but different densities to analyze spontaneous segregation [13,14,15]. The particle types chosen for this investigation [16] are glass and polypropylene. Although the density difference is required, the comparably high density of glass has the disadvantage of inducing beam hardening at a non-negligible level, which hampers the segmentation of the different materials. Another difficulty is the presence of three different materials (glass, polypropylene and air) with quite different densities. Thus, other than in former UFXCT studies, the available dynamic range to differentiate between each of them is reduced. While the glass particles can still be distinguished quite effectively from the other materials, the contrast between polypropylene particles on the one hand and artifacts and noise in the air region on the other hand is rather low. Therefore, the beam hardening correction procedure described here mainly aims at improving the distinguishability between polypropylene particles and air.

As beam hardening is also a relevant source of error in medical and industrial applications, a number of mitigation strategies have been developed. In medical CT, the presence of metals, such as implants [17] or dental fillings [18], causes severe artefacts, and different metal artefact reduction strategies are proposed [17,19]. Besides this, even the density difference between bone and tissue leads to beam hardening errors. These are less severe, but cause problems in the developing field of quantitative CT in medical applications [20,21]. In nondestructive testing, high-density materials are much more common, and beam hardening is thus even more present. However, there is also more tolerance in terms of radiation dose and other technical issues. For instance, dual-energy measurements can be applied to gain more information about the energy-dependent attenuation [22,23].

Typically, the complexity of beam hardening correction strategies depends on the complexity of the object of investigation. For the special case of single-material applications, a simple calibration with different material thicknesses can be applied. Theoretically, this approach can be extended to more materials [24], but with an exponentially growing effort on calibration data generation and storage, as each material length combination needs to be measured in advance. In general, however, more sophisticated methods are required. Most of them focus on the segmentation of the different materials followed by the simulation of beam hardening [25]. Instead of segmented data, available geometry data on, e.g., metal parts can likewise be used [17]. The problem with this strategy is the knowledge required on the energy-dependent attenuation coefficients of all involved materials, along with the computational effort required for the simulation. Additionally, X-ray scattering is intrinsically tied to beam hardening as it also influences the X-ray energy spectrum and has to be considered as well to ensure highly accurate results [23,26]. Including scattering into the simulation leads to even more extensive calculations, due to its stochastic nature and the variety of possible beam paths. Neglecting it, however, leads to inaccurate results, despite the accuracy of the energy-dependent attenuation coefficient data. To reduce complexity, a method that uses a limited number of energy bins for the attenuation coefficient along with minimizing the difference between the measured and the simulated sinogram is proposed in [27]. A further simplification is presented in [28], where different combinations of a monoenergetic forward projection of the segmented image and the original measured sinogram are used to optimize the resulting reconstructed images with respect to flatness. A similar approach is described in [29], where, instead of a segmented image, the reconstructed image and a version of it modified by histogram deformation are forward-projected to build different combinations of sinograms. The resulting set of reconstructed images was then likewise subject to optimization regarding flatness. Finally, deep learning-based methods have also been successfully applied to reduce beam hardening artefacts [20,30]. From all these approaches one can draw the conclusion that, with sufficiently high effort, beam hardening can be reduced successfully and in many applications. Consequently, the focus of recent research on this topic lies more on simplifying the algorithms to be applied than on improving the result. For the present application, the number of materials and thus the complexity of the beam hardening problem and the variety of the solution space is limited. Therefore, an approach is presented that combines segmentation methods with calibration methods and works without knowledge of the energy distribution of the X-ray source or the energy-dependent X-ray attenuation of the materials involved.

## 2. Granular Mixing Experiments

### 2.1. Experimental Setup

**Figure 1:**
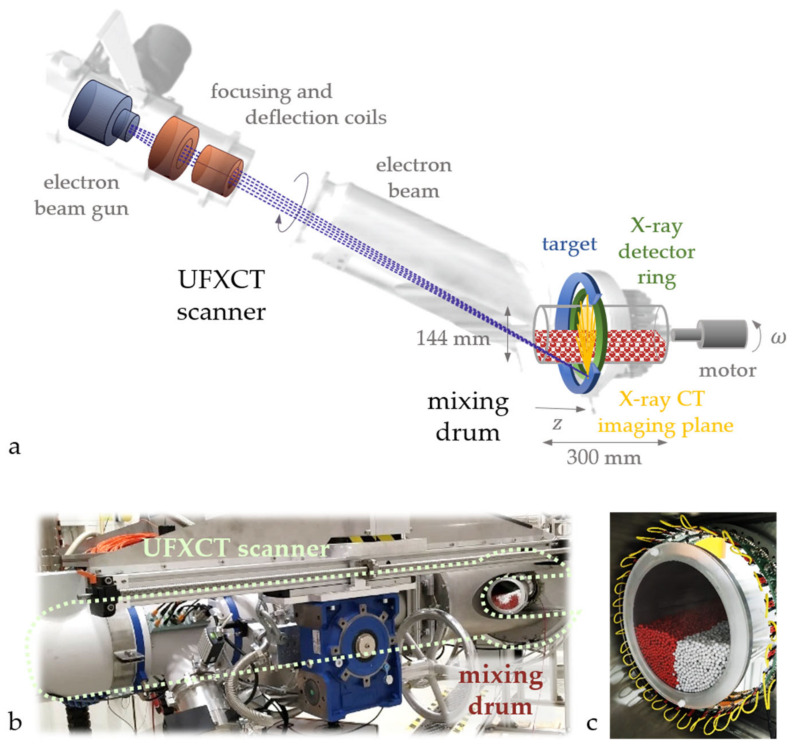
Experimental setup depicted as (**a**) a schematic of the mixing drum with the particle bed and the UFXCT system, including the electron beam generation and deflection part on the left and the X-ray CT imaging part on the right, (**b**) a photograph of the UFXCT scanner and the inserted mixing drum after one rotation, and (**c**) a photograph of the particle bed in its initial segregated state surrounded by the detectors of the UFXCT system.

The experimental study motivating the application of a beam hardening correction algorithm concerns the binary mixing of particles in a cylindrical drum [16]. The experimental setup (Figure 1a) consists of a drum made of polymethyl methacrylate (PMMA) with an inner diameter of 144 mm and a length of 300 mm, which can rotate with a speed of up to 45 rpm. The particles to be mixed are spherical and have the same size, i.e., a diameter of 4 mm, but different densities. Polypropylene (PP) particles with a density of 0.9 g/cm^3^ and glass particles with a density of 2.5 g/cm^3^ form the particle bed at a volumetric species ratio of 1:1. The filling level of the drum is varied between the experiments, but only results for a filling level of 0.5 are shown in this paper. For further experimental results, we refer to [16].

### 2.2. Computed Tomography System

Tomographic projections are gathered by an ultrafast X-ray computed tomography (UFXCT) scanner. In contrast to former applications, the scanner is turned to a horizontal position (Figure 1a,b) to provide a vertical tomographic imaging plane. UFXCT reaches imaging rates of up to 8000 fps by using electron beam deflection instead of mechanically rotating components. The electron beam is focused onto a tungsten target and deflected with high frequency along a partially circular path on the target to generate an X-ray source that is rapidly rotating around the object of investigation. A statically mounted X-ray detector ring comprising 256 detecting elements, which are read out in parallel and sampled at a frequency of 2 MHz, synchronously captures tomographic projections of the object. To cope with this large amount of data, it is streamed to a PC via Gigabit Ethernet connections. Image reconstruction based on the filtered back-projection algorithm is performed in real-time on GPUs using a data pipeline scheme [31]. However, due to the short integration time, the measured values are rather noisy, which leads to a reduced image quality compared to conventional CT images.

### 2.3. Measuring Procedure

Prior to each experiment, the particles are sprayed with a propan-2-ol aerosol to prevent static electric charges from disturbing the mixing process. Initially, the drum is filled with both particle species in a completely separated state (see Figure 1c). This is ensured by inserting a vertical sheet in the center of the drum prior to filling and removing it before conducting the experiments. The rotation of the drum is started shortly after the X-ray data acquisition is initiated to ensure the whole mixing process is captured. A further advantage of this procedure lies in a certainnumber of images of the separated bed at rest at the beginning of the measurement, which can be averaged to get less noisy data for the beam hardening calibration, as described in Section 3.3. Tomographic projections are then recorded in single-plane mode at a rate of 1000 fps, whereas every 10 frames are averaged for an improved signal-to-noise ratio with negligible motion blurring.

## 3. Simplified Beam Hardening Correction Algorithm

### 3.1. Idea

Without knowledge about the energy distribution of the initial and the detected X-ray radiation and the exact energy dependency of the X-ray attenuation coefficients of the particles and the drum, the exact reconstruction of the material distribution is a largely ill-posed inverse problem. In order to still be able to improve the result compared to the general monoenergetic CT image reconstruction, a simplified model of beam hardening is proposed for its correction. It does not claim to completely compensate, but to strongly alleviate the beam hardening effect so as to facilitate a trustful image post-processing. We assume the beam hardening effect of the denser material, in our case glass particles, to be significantly higher than that of the other materials, and thus neglect the latter. The idea is now to segment the glass particle region in the conventionally reconstructed images, which is quite reliable due to the contrast conditions in our material distribution, and use their forward projection to correct the measured projections by removing the nonlinear share of the extinction. To quantify the nonlinearity between the thickness of the irradiated glass within the particle mixture and the measured extinction, a calibration procedure based on beams passing only glass particles is conducted. Theoretically, this method can be adapted to other applications that include one material of significantly higher density by customizing the calibration procedure.

### 3.2. Mathematical Description

The attenuation of monoenergetic X-ray intensity I along a spatial coordinate *x* can generally be described using Beer–Lambert’s law as

(1) $$I\left(X\right)=I\left(0\right)exp\left(-\int_{0}^{X}\mu \left(x\right)dx\right),$$

wherein μ is the linear attenuation coefficient and the exponent is usually referred to as extinction E, i.e.,

(2) $$E_{Mono}\left(X\right)=ln\left(I\left(0\right)/I\left(X\right)\right)=\int_{0}^{X}\mu \left(x\right)dx.$$

In the case of distinct materials, the integral becomes a sum of products of the linear attenuation coefficients and their respective effective length L along the beam path. In our case it can be written as

(3) $$E_{Mono}\left(X\right)=E_{Mono}\left(L_{PMMA}\right)+E_{Mono}\left(L_{PP}\right)+E_{Mono}\left(L_{G}\right)\;=\mu_{PMMA}L_{PMMA}+\mu_{PP}L_{PP}+\mu_{G}L_{G},$$

with PMMA being the material of the drum and polypropylene (PP) and glass (G) the materials of the particles. For polyenergetic X-ray beams, this model can serve as an approximation of the real attenuation, as mentioned above. Therein, μ is replaced by the mean linear X-ray attenuation coefficient $\bar{\mu }$ for the polyenergetic radiation in use. This simplified model, however, ignores the beam hardening effect, i.e., a higher attenuation of X-ray photons of lower energy, which occurs due to the dependency of μ on the photon energy. This leads to a reduction in the effective $\bar{\mu }$ along the material length l and results in a nonlinear relationship between irradiated material length and extinction. If this effect is taken into account at least for the glass, as it is far more pronounced than in plastic, where linearity mainly holds, Equation (3) changes to

(4) $$E_{Poly}\left(X\right)=\bar{\mu_{PMMA}}L_{PMMA}+\bar{\mu_{PP}}L_{PP}+\int_{0}^{L_{G}}\mu_{G}\left(l\right)dl.$$

As we do not know the characteristics of $\mu_{G}\left(l\right)$ apart from the general condition $\int_{0}^{L_{G}}\mu_{G}\left(l\right)\;dl<\bar{\mu_{G}}L_{G}$, Equation (4) can be rewritten as

(5) $$E_{Poly}\left(X\right)=\bar{\mu_{PMMA}}L_{PMMA}+\bar{\mu_{PP}}L_{PP}+E_{Poly}^{\left(G\right)}\left(L_{G}\right),$$

with $E_{Poly}^{\left(G\right)}\left(L_{G}\right)$ being the nonlinear extinction function of pure glass that needs to be determined through calibration.

### 3.3. Calibration

**Figure 2:**
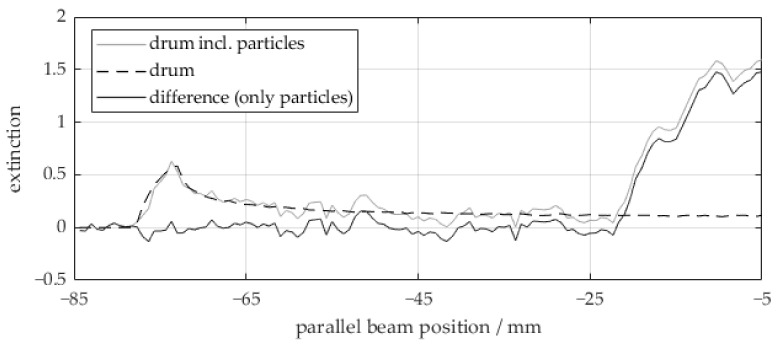
Subtraction of the simulated extinction of the drum wall from the measured extinction of the drum including particles to obtain the extinction of the particles. It is depicted for one half of a parallel beam projection at an arbitrary angular position, wherein the parallel beam position 0 corresponds to the center of the drum (compare Figure 3a).

For the calibration of the beam hardening correction algorithm, only the function $E_{Poly}^{\left(G\right)}\left(L_{G}\right)$ needs to be characterized, as we assume the extinction of the glass to be the only nonlinear share in the measured extinction. For this purpose, measured extinction values for different values of $L_{G}$ are required. Theoretically, this can be done by calibration measurements of different but known material thicknesses. However, in our application, the calibration data can be directly derived from the measured sinogram data. Thus, no additional calibration measurements are needed. Instead, calibration data are inherently gained for each measurement at the same conditions without temporal distance or changes in the setup. Following Equation (5), we can retrieve the extinction of the glass region by subtracting the extinctions of the drum and the polypropylene particles from the measured extinction. For the drum extinction, a monoenergetic forward projection of the known drum region has been performed, using its mean attenuation coefficient from the conventionally reconstructed CT images. As can be seen in Figure 2, this forward projection fits the drum extinction in the measured extinction curve quite well, and hence is suitable for removing the extinction of the drum.

**Figure 3:**
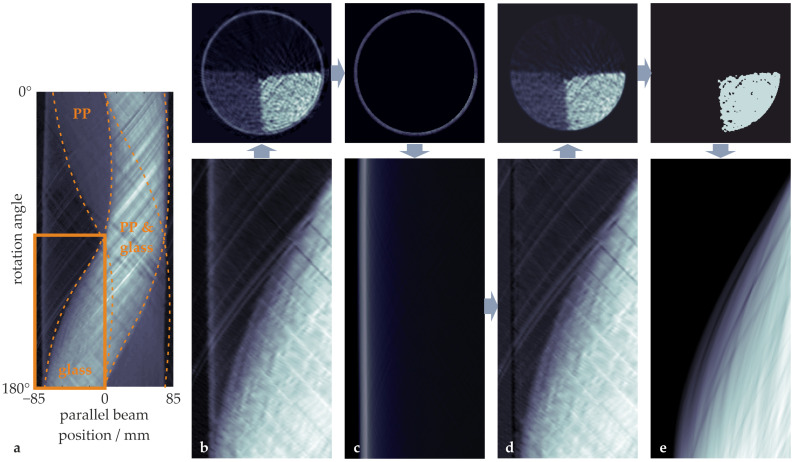
Cross-sectional images of different components of the experimental setup in combination with the part (specified in (**a**)) of the corresponding parallel beam sinograms that include the glass region, but no polypropylene particles: (**b**) drum with particles, (**c**) drum without particles, (**d**) particles without drum ((**d**) = (**b**) – (**c**)), and (**e**) segmented glass particles. The arrows indicate the direction of processing.

For the extinction of the polypropylene particles, this segmentation-based approach is not suitable, as this segmentation is the aim of the whole analysis, which should be improved by the beam hardening correction. Instead, measured extinction values for rays with $L_{PP}=0$ are sought in the sinograms. As the tomographic imaging of our granular mixing experiments starts with completely separated particle distribution, a sufficient number of such projections can be found for different $L_{G}$ values in the respective sinograms (Figure 3a). As the rotation of the drum is started with a short temporal offset, the CT data sets contain about 100 sinograms with the particle bed at rest. By using the average of those sinograms (Figure 3b), the statistics and accuracy of the calibration can be further improved.

**Figure 4:**
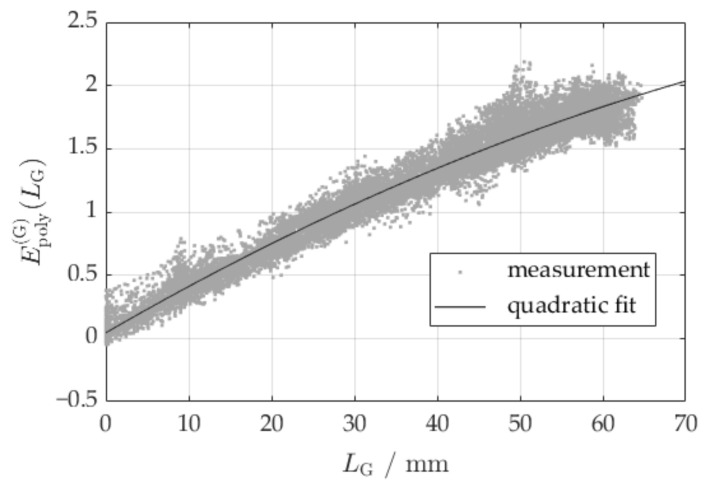
Relationship between material thickness $L_{G}$ and measured extinction of the glass particles including a quadratic fitting function.

**Figure 5:**
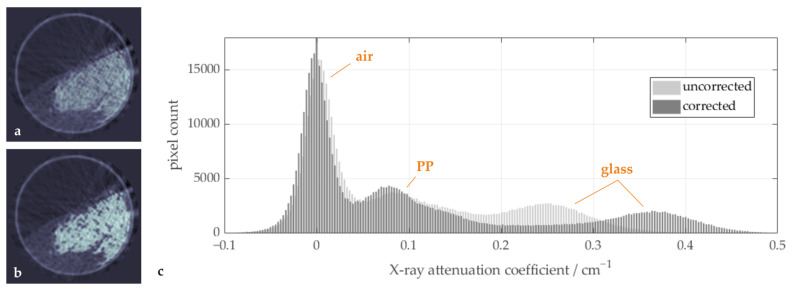
Sample images (**a**) before and (**b**) after the beam hardening correction and (**c**) histograms showing the distribution of the X-ray attenuation coefficient for both image stacks.

For all the selected projections, i.e., those without polypropylene particles, the respective drum extinction (Figure 3c) is subtracted and the resulting extinction values (Figure 3d) are plotted as a function of the length $L_{G}$ (Figure 4). This length has been derived using the monoenergetic forward projection of the binary image representing the glass particles (Figure 3e), which in turn has been gained through segmentation of the originally reconstructed image. Segmentation has been performed by thresholding the reconstructed image at a linear attenuation coefficient corresponding to the respective local minimum position in the histogram of the reconstructed images (compare Figure 5c). As expected, the resulting relationship of the measured extinction $E_{Poly}^{\left(G\right)}\left(L_{G}\right)$ and the actual material length $L_{G}$ is nonlinear, as can be seen in Figure 4.

By approximating the nonlinear extinction function $E_{Poly}^{\left(G\right)}\left(L_{G}\right)$ as a quadratic function, we can write

(6) $$E_{Poly}^{\left(G\right)}\left(L_{G}\right)=a{L_{G}}^{2}+\bar{\mu_{G}}L_{G}+c.$$

The coefficients a and c are obtained from the calibration data in Figure 4 by regression using a second order polynomial function. Other nonlinear functions may be chosen as the approximation for $E_{Poly}^{\left(M\right)}\left(L_{M}\right)$ for a dense material M other than glass, if this polynomial function does not fit the calibration data.

### 3.4. Beam Hardening Correction Procedure

To compensate the beam hardening effect caused by the glass beads, the nonlinear share of Equation (6) has to be subtracted from the measured sinograms. To determine the length $L_{G}$ and the correction values, a similar strategy as used during the calibration is followed. First, the share of the attenuation from the drum is subtracted from the measured extinction values $E_{Poly}\left(X\right)$. Second, the region of the glass beads in the reconstructed images is segmented by applying the threshold given above. The forward projection of this region gives the length $L_{G}$ for all projections. Third, given the coefficients a and c from the calibration, the quadratic and offset term from Equation (6) are subtracted from the sinogram. All steps combined result in

(7) $$E_{\mathrm{Corr}}\left(X\right)=E_{\mathrm{Poly}}\left(X\right)-\bar{\mu_{PMMA}}L_{PMMA}-a{L_{G}}^{2}-c.$$

Finally, the corrected extinction values $E_{\mathrm{Corr}}\left(X\right)$ are used for reconstructing the improved images of material distribution. To ensure the validity of the method, the procedure may be repeated iteratively from the second step.

## 4. Results and Discussion

By applying one iteration of the described beam hardening correction algorithm to the tomographic projection data of the mixing drum experiment, improved cross-sectional CT images are retrieved. As can be seen in Figure 5, the contrast between the particle species is increased and image artifacts are reduced (b) compared to a standard filtered back-projection image (a). In the histogram of both image stacks (c), further improvements are observed: the peaks for air and polypropylene are narrower after the beam hardening correction and the valley between the peaks of polypropylene and glass is broader and lower. Another iteration of the correction algorithm only led to marginal differences in the reconstructed image as well as the histogram, and was thus omitted in the further analysis.

**Figure 6:**
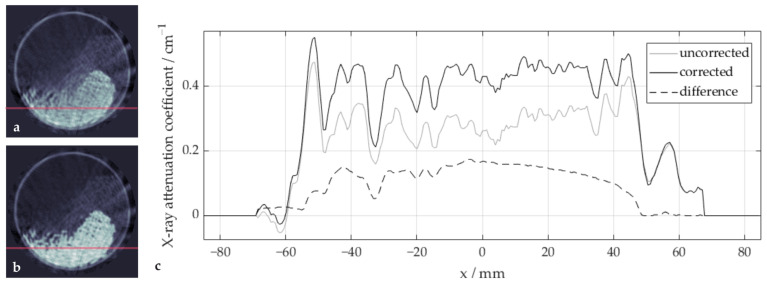
Effects of the beam hardening correction in the glass region: sample image (**a**) before and (**b**) after the beam hardening correction along with (**c**) corresponding profiles of a selected image line/row (marked in red).

To demonstrate the effect of the correction algorithm within the glass region in more detail, a sample image with high glass content is shown in Figure 6 with (b) and without (a) beam hardening correction, together with a profile of the reconstructed X-ray attenuation coefficients along a horizontal image line (c). Without correction, the X-ray attenuation coefficients in the center of the glass sphere bulk are lower than in the outside regions. This bathtub-shaped curve is a typical effect of beam hardening in homogeneous regions of high absorbing materials, as the underestimation of the extinction increases with material length and is therefore more pronounced in the center region. Note that the profiles include gaps and polypropylene particles, which should be ignored when assessing the shape of the profiles. After the correction, the profile is flattened, and the difference between both profiles clearly shows the described, in this case inverse bathtub, shape.

**Figure 7:**
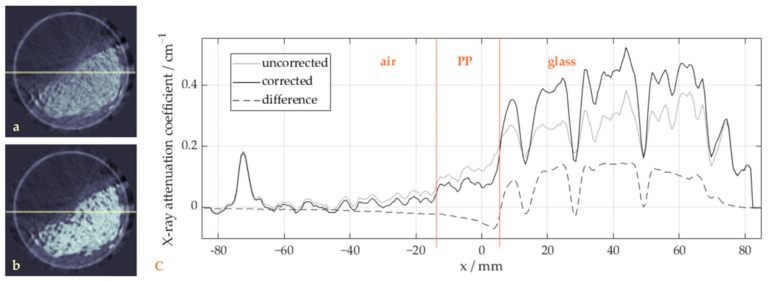
Effect of the beam hardening correction outside the glass region: sample images (**a**) before and (**b**) after the beam hardening correction along with (**c**) corresponding profiles of a selected image line/row (marked in red).

Figure 7 similarly shows a sample image, which in this case focuses on the effects of the beam hardening correction outside the glass region. Beside the reduction in visible artefacts in the air region outside the particle bulk, the X-ray attenuation coefficient profiles show important improvements. Within the polypropylene particle region as well as in the surrounding air, the curve is flattened compared to the slightly inclined curve without correction. This artificial inclination results from inconsistencies between projections from different directions due to beam hardening, which are spread into neighboring regions during the reconstruction process. The reverse shape in the difference curve also illustrates how parts of the measured attenuation shift from the glass towards the outer regions. At the transition between each of the materials, a clear step can be observed after correction, which improves the differentiation between the materials during segmentation.

**Figure 8:**
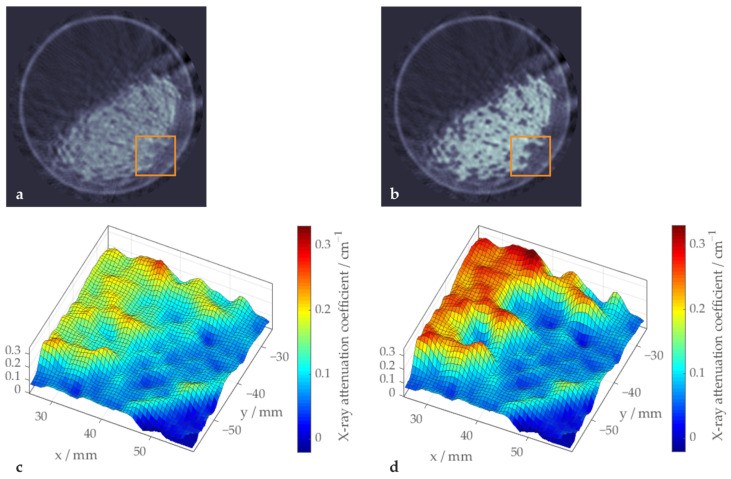
Sample image illustrating the improved homogeneity of the polypropylene region achieved with beam hardening correction (**b**,**d**) compared to the uncorrected image (**a**,**c**). The orange squares in (**a**,**c**) indicate the sections shown in (**c**,**d**).

This effect becomes even more obvious when looking at a small region of the particle bed depicted as a landscape, as shown in Figure 8. This example shows two indentations of polypropylene particles into the glass particle core. Though clearly recognizable as such, even in the uncorrected image, the reconstructed attenuation coefficient in this case is noticeably higher than in the outer, ring-shaped polypropylene region.

With beam hardening correction, these indentations show much lower attenuation coefficients, lying in the same range as outside the glass particle core. Furthermore, the contrast between glass and polypropylene is perceivably higher and the homogeneity of both materials is improved. Note that both landscape illustrations use the same scale of attenuation coefficient values.

**Figure 9:**
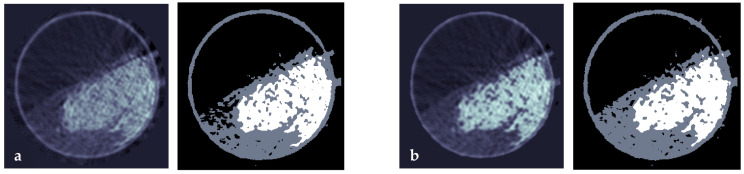
Effect of the beam hardening correction on the segmentation of the gas region from the particle region in the (**a**) uncorrected and (**b**) beam hardening corrected version of the reconstructed image and final three-phase segmentation of the corrected image.

Finally, Figure 9 shows how the beam hardening correction affects the segmentation of the polypropylene particle region from the gas region. The gray value threshold for the segmentation of the uncorrected as well as the corrected image was chosen according to the respective minimum between the air peak and the polypropylene peak in the attenuation coefficient histograms in Figure 5c. In the uncorrected image, the polypropylene region contains inhomogeneities, which are not quite obvious in the gray value images but lead to large differences in void fraction distribution in the segmented image. With beam hardening correction, the differences in the local mean attenuation coefficient for polypropylene are smaller, and thus the resulting void fraction is more homogenous. Thus, the value and applicability of this simplified beam hardening correction algorithm could be successfully demonstrated.

## 5. Summary and Conclusions

In this paper, we have presented a simplified beam hardening correction algorithm, which is suitable for computed tomography imaging of material distributions with a limited number of materials of different densities. The algorithm focusses on the densest material present in the object of investigation and its share in beam hardening. It takes advantage of the comparably smaller contributions of the other materials to beam hardening, which are neglected in this case. Thus, this simplified beam hardening correction algorithm combines the advantages of simulation-based methods with the simplicity of calibration-based methods. On the one hand, it requires low effort, as no energy-resolved measurements or simulations are needed. On the other hand, the nonlinearity of the extinction is successfully captured and used to reduce beam hardening artefacts significantly. The positive effects of this beam hardening correction algorithm have been analyzed using ultrafast X-ray CT measurements of a rotating drum, in which polypropylene and glass particles are mixed. It could be shown that the different materials show narrower frequency distributions in the attenuation coefficient histogram, and the homogeneity between different regions of the same material was higher than without beam hardening correction. The contrast between the different materials was increased. Thus, the materials could better be distinguished from each other, which was the main goal of the beam hardening correction, and this has been demonstrated with the exemplarily segmented images. Negative effects due to the neglected contribution of the lower-density materials to beam hardening were not observed. Early results have already been obtained with our method, and are promising in terms of investigating mixing in a cylindrical drum as a function of time. Discussions of the experimental results of particle mixing would go beyond the scope of this paper, and will appear in upcoming publications [16].

## Data Availability

The data presented in this study are openly available in RODARE at https://doi.org/10.14278/rodare.2241.
